# Short-term effect of zoledronic acid upon fracture resistance of the mandibular condyle and femoral head in an animal model

**DOI:** 10.4317/medoral.18449

**Published:** 2013-03-25

**Authors:** Fabio Camacho-Alonso, Pía López-Jornet, Ascensión Vicente-Hernández

**Affiliations:** 1 Full Professor of Oral Medicine. University of Murcia (Spain); 2 Contracted Doctor of Orthodontics. University of Murcia (Spain)

## Abstract

Objective: The aim of this study was to compare the effects in terms of resistance to fracture of the mandibular condyle and femoral head following different doses of zoledronic acid in an animal model. Study design: A total of 80 adult male Sprague-Dawley rats were included in a prospective randomized study. The animals were randomly divided into four groups of 20 rats each. Group 1 (control) received sterile saline solution, while groups 2, 3 and 4 received a accumulated dose of 0.2 mg, 0.4 mg and 0.6 mg of zoledronic acid, respectively. The animals were sacrificed 28 days after the last dose, and the right hemimandible and the right femur were removed. The fracture strength was measured (in Newtons) with a universal test machine using a 1 kN load connected to a metal rod with one end angled at 30 degrees. The cross-head speed was 1 mm/min. Later, the specimens were observed under a scanning electron microscope with backscattered electron imaging (SEM-BSE). At last, chemical analysis and elemental mapping of the mineral bone composition were generated using a microanalytical system based on energy-dispersive and X-ray spectrometry (EDX). Results: A total of 160 fracture tests were performed. The fracture resistance increased in mandible and femur with a higher accumulated dose of zoledronic acid. Statistically significant differences were recorded versus the controls with all the studies groups. The chemical analysis in mandible showed a significantly increased of calcium and phosphorous to compare the control with all of the study groups; however, in femur no statistically significant differences between the four study groups were observed. Conclusions: The administration of bisphosphonates increases the fracture resistance in mandible and femur.

** Key words:**Zoledronic acid, bisphosphonates, animal experimentation, fracture test.

## Introduction

The mechanical properties of bone directly condition fracture risk and are the best indicators of bone strength (1-7). Bisphosphonates decrease bone resorption, increase bone mineral density and decrease the risk of fracture.

Zoledronic acid is a nitrogen-containing, highly potent bisphosphonate, and is commonly used as supportive treatment for cancer patients. Currently, it has also been approved in a single dose/year regimen for the treatment of postmenopausal osteoporosis. The zoledronic acid and other oral and intravenous bisphosphonates are associated with osteonecrosis of the jaw. This type of osteonecrosis is limited to the craniofacial region and its physiopathology is not known (2,8).

The specific pharmacological properties of bisphosphonates include selective uptake in the skeleton, preferentially at sites of active bone remodeling, where they decrease bone resorption mainly through direct effects upon the osteoclasts.

The high bone turnover rate in the jaw, probably a consequence of its high mechanical load, may lead to an increased local uptake of bisphosphonate and thus contributing to the development of osteonecrosis in this location (1,3,9). Although some authors like Baus et al. (5) have shown that in rats given different doses of ibandronate, the uptake of this bisphosphonate in the mandible is relatively similar to that seen in other bones of the skeleton.

Although the modifications in bone hardness after bisphosphonates administration have been systematically documented, the cause of such changes remains unclear (9). Determining the cause of lessened resistance is becoming particularly relevant in the light of the recent increase in the incidence of atypical femoral fractures presenting features consistent with reduced bone resistance (1,7,8).

In this sense, the objective of the present study was to compare the effects upon fracture resistance of the mandibular condyle and femoral head with different doses of zoledronic acid in an animal model, and analyze the mineral composition of these bones.

## Material and Methods

-Experimentation animals 

The animals used in this study were obtained from the Experimentation Animals Unit (Support for Research Unit) from University of Murcia (Spain).

A total of 80 adult male Sprague-Dawley rats with a mean weight of 250 g (range 210-270 g) were included in this prospective randomized study, following a protocol approved by the Bioethics Committee of the University of Murcia (Spain). The study was carried out between January and September 2010. The animals were individually housed in plastic cages in a monitored environment (21º C and 12: 12 hours light: darkness cycle). The rats were acclimatized for one week before the start of the study, and had free access to drinking water and a standard laboratory rat food pellet diet. The entire study was carried out in abidance with the corresponding European Union guidelines.

The animals were randomly divided into four groups of 20 rats each. Group 1 (control) received sterile saline solution, while groups 2, 3 and 4 received a accumulated dose of 0.2 mg, 0.4 mg and 0.6 mg of zoledronic acid, respectively; through a single weekly intraperitoneal dose consisted in zoledronic acid of 0.2 mg/250 g ( Table 1). The amount of drug to be administered was extrapolated from the amount of drug received by patients for cancer-related bone disease (4).

**Table 1:**
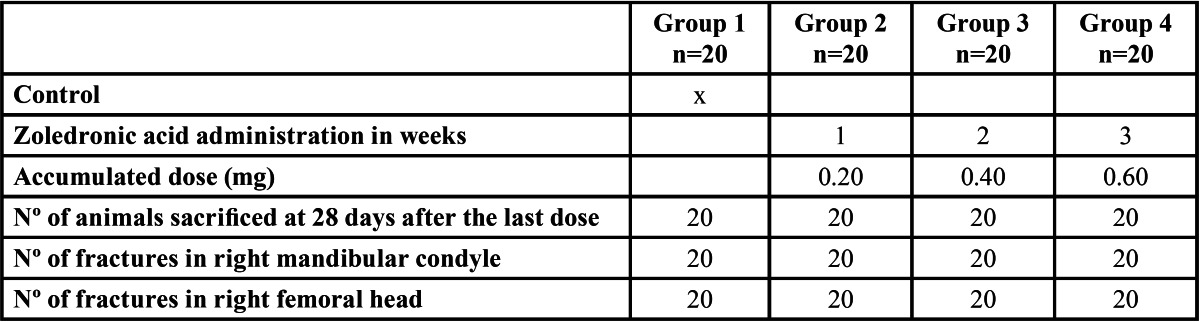
Study design (n = 80 male Sprague-Dawley rats).

The animals were sacrificed with carbon dioxide 28 days after the last dose. Immediately after sacrifice, the right hemimandible and femur were removed.

Samples for biomechanical testing were wrapped in saline-soaked gauze and frozen at -20ºC until testing. The samples were posteriorly thawed at room temperature and immersed in saline solution 5 minutes before testing to allow temperature equilibration.

-Fracture resistance (biomechanical testing procedure)

Specimens from the mandible and femur were mounted in a 40 mm long copper cylinder with an internal diameter of 30 mm, inserting half the length in plaster. The fracture strength of the hemimandibular condyle and the femoral head was measured (in Newtons) using a universal test machine (Autograph AGS-1KND, Shimadzu, Kyoto, Japan) with a 1 kN load connected to a metal rod with one end angled at 30 degrees. The crosshead speed was 1 mm/min.

-Study of the samples with the scanning electron microscope

The samples were processed (through carbon bath) for their observation to the scanning electron microscope (SEM-BSE) (Oxford Instruments INCA 300 EDX System, Abingdon, Oxfordshire, Reino Unido). All observations were carried out to a distance of 19 mm, a voltage of 15 kv and a 15 × magnification.

-Chemical analysis of the mineral bone composition (microanalysis of elements)

**Figure 1:**
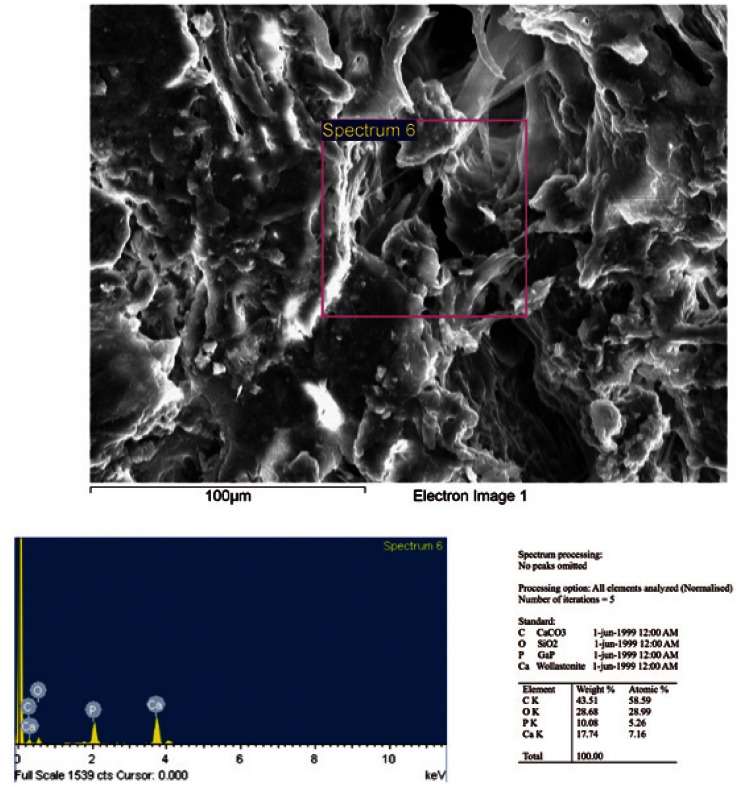
Chemical microanálisis of the mineral bone composition based on energy-dispersive and X-ray spectrometry (EDX), showing a section of bone using scanning electron microscope, and the spectrum of elements in the sample.

At last, we perform an analysis of the mineral bone composition using a microanalytical system based on energy-dispersive and X-ray spectrometry (EDX) (Fig. 1). This system allows evaluating the relative concentration of all the chemical elements present in the bone, using a timely analysis and elemental mapping to determine the mineral distribution. The relative concentration of each element is indicated by a color scale, where for each element, the dark blue color indicates the absolute zero and white indicates the concentration absolute 100% of that element.

-Statistical analysis

The data were analyzed using the SPSS version 12.0 statistical package (SPSS® Inc., Chicago, IL, USA). The study was designed with an 80% statistical power, assuming security of 95%. A descriptive study was made of each variable. The Kolmo-gorov-Smirnov normality test and Levene variance homogeneity test were applied, and the data showing a skewed distribution were analyzed using a nonparametric ranking test. We used the Mann-Whitney U-test (for two independent samples), for quantitative variables. Statistical significance was accepted for p£0.05.

## Results

A total of 160 fracture tests were performed (80 in mandible and 80 in femur). The fracture resistance increased in mandible and femur with a higher accumulated dose of zoledronic acid. Statistically significant differences were recorded versus the controls with all the studies groups ( Table 2).

**Table 2:**
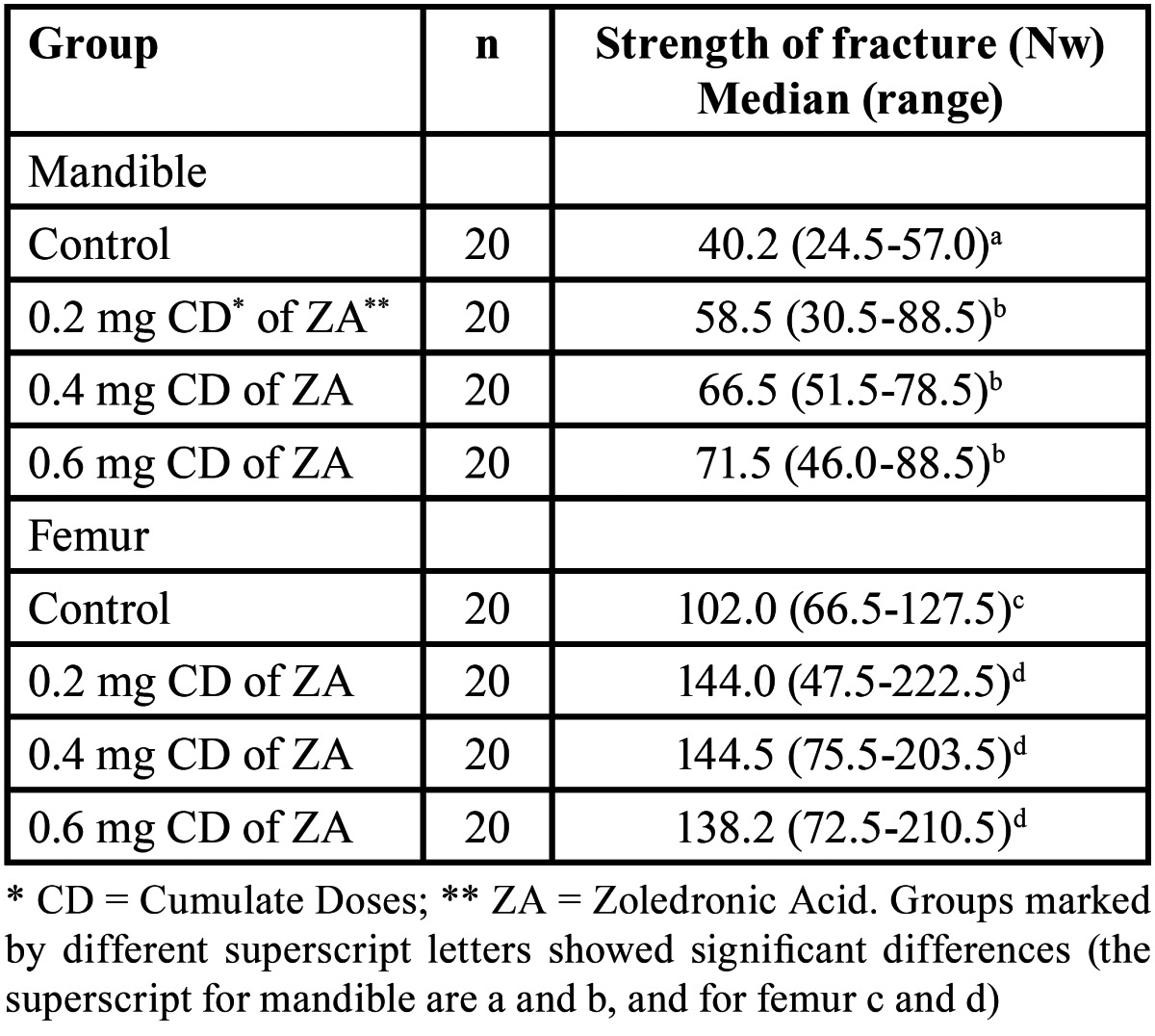
Results of the bone fracture tests (Mann-Whitney U test).

**Figure 2:**
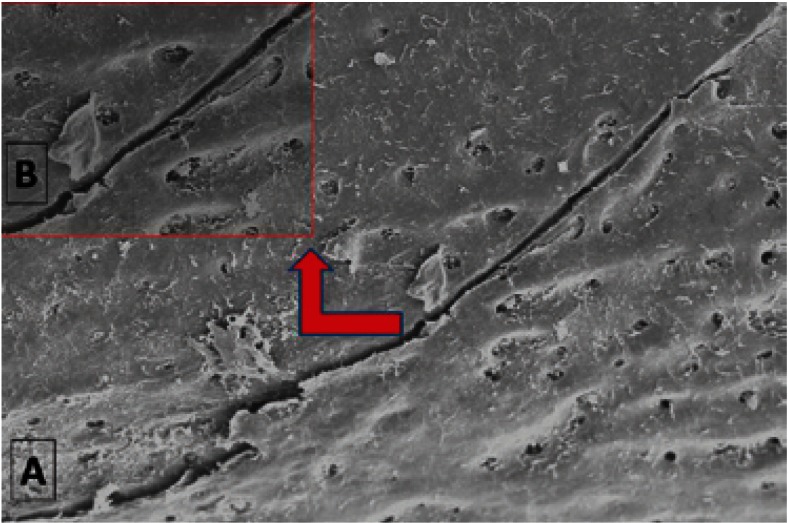
A) Mandibular microfracture next to the central fracture zone in the mandibular condyle, using scanning electron microscope. B) Mandibular microfracture with greater mafgnification.

**Figure 3:**
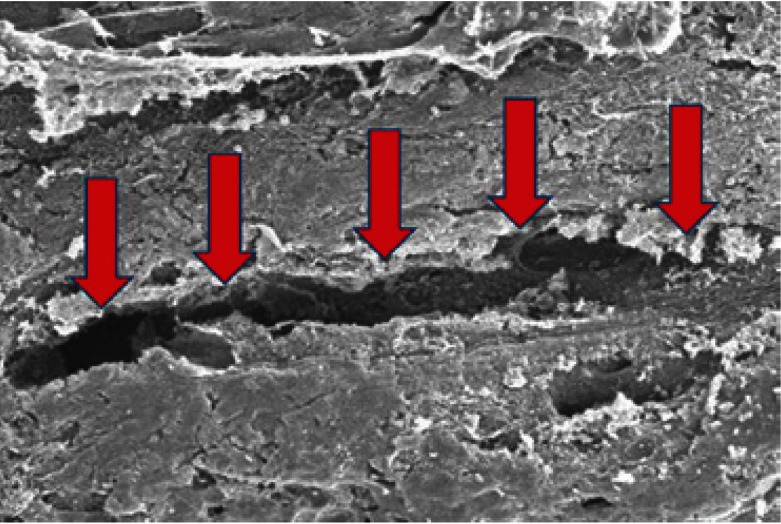
Microfracture in femur next to the central fracture zone in the femoral head.

The observation of specimens under a scanning electron microscope, once produced the bone fracture in the mandibular condyle, showed that 5 of the 20 samples controls (25%) had at least one microfracture next to the central fracture zone in the mandibular condyle (Fig. 2); while in groups where zoledronic acid was administered was not observed any microfracture adjacent to the central fracture zone. Similarly, in the samples where the bone fracture was made in the femoral head, 6 of 20 samples controls (30%) had microfractures next to the central fracture zone in the femoral head (Fig. 3), while there were no microfractures in the study groups.

The chemical analysis in mandible showed a significantly increased of calcium and phosphorous to compare the control with all of the study groups; however, in femur no statistically significant differences between the four study groups were observed ( Table 3).

**Table 3:**
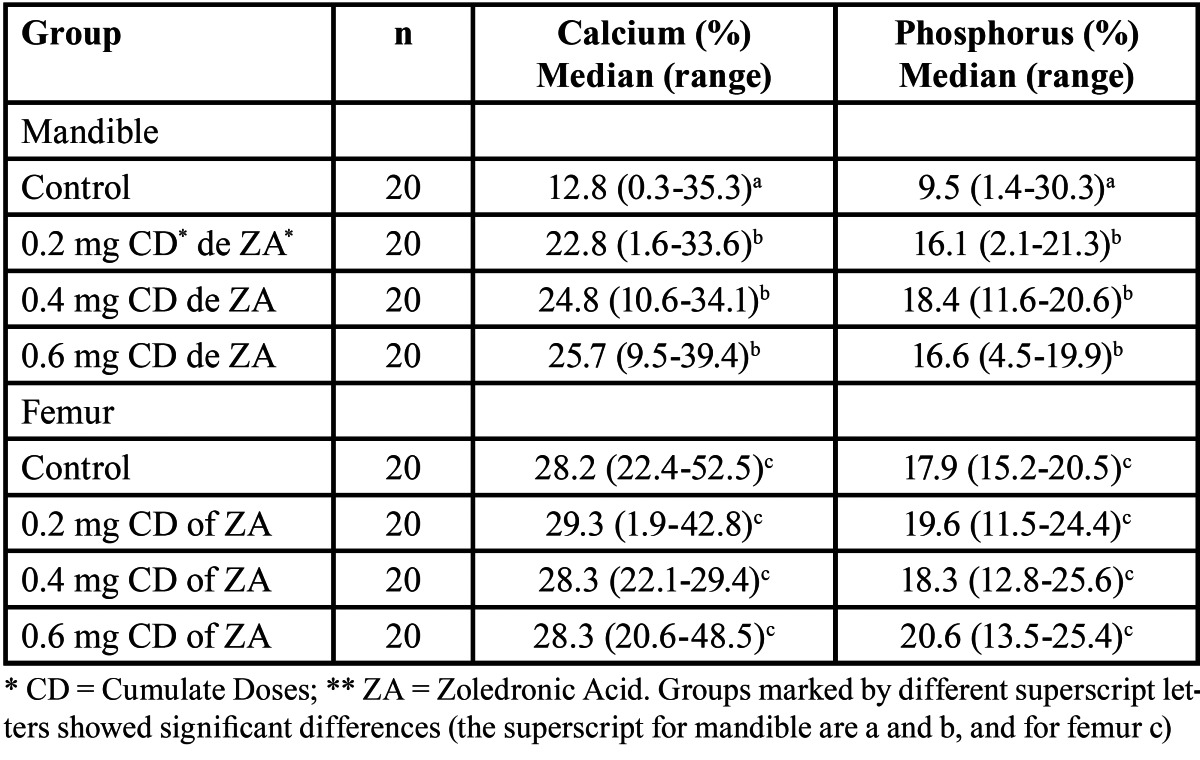
Relative concentration of calcium and phosphorus based on measurement of energy-dispersive and X-ray spectrometry by scanning electron microscope (Mann-Whitney U test).

## Discussion

The mechanical properties of bone directly condition fracture risk and are the best indicators of bone strength. The bisphosphonates, with different degrees of potency and efficacy, prevent the loss of bone mineral mass and density In our study, the biomechanical testing showed the short-term administration of zoledronic acid in rats to increase the resistance to fracture of both mandibular condyle and femoral head; and the chemical analysis showed an increase in the levels of calcium and phosphorus in both bones, which was higher as increasing the cumulative dose of the bisphosphonate.

Comparing studies investigating methods on the mechanical properties of bone is not easy. Mechanical properties are additionally influenced by a variety of factors, such as anatomical location, specimen size and shape, loading mechanism, and displacement time of the load (7-10).

The evaluation of these biomechanical properties of bone is very important for assessing changes in bone mass and architecture. However, according to Shahnazari et al. (9), standardized testing protocols are not available, and the bone samples used are derived from different species with different specimen geometries types (whole bones versus machined specimens, cortical versus trabecular specimens), and different preservation fluids involving different concentrations are moreover used.

Only in animals or material from cadavers can destructive analytical techniques be used, such as the triple-point flexion test or vertebral body resistance to compression, based on universal material analyzing systems and specific software applications. Basically, the aim is to quantify structural parameters such as bone resistance to fracture (maximum load or fracture load). In our study testing was carried out under conditions of controlled displacement at a crosshead speed of 1 mm/min. The objective of this speed was to avoid any dynamic effect, in accordance with other authors (8-10).

In recent years there has been an important increase in the number of experimental models developed to understand the mechanisms by which bisphosphonates exert preventive effects against skeletal complications in malignant diseases characterized by the development of bone metastases (11,12). Reductions in fracture risk in bisphosphonate-treated patients are assumed to be the result of improved or maintained bone biomechanical properties. However, there is concern that an exaggerated increase in bone mass not accompanied by improvement in bone architecture may affect the quality of bone. Increased biomechanical properties of the whole bone are partially offset by adverse changes in the bone material (7). In addition, one of the limitations of the biomechanical in rodent studies is the difficulty to extrapolate the results to human beings, due to the differences in bone metabolism of both species.

Bisphosphonates have specific pharmacological properties that differentiate them from all other bone resorption inhibitors, including long-term retention within the skeleton and persistence of their effects after the discontinuation of treatment (2,3,13-16). This effect would keep properties improved biomechanical of bone, at least in the short term of time after stopping use.

In our study, the administration of bisphosphonates in Sprague-Dawley rats, increased the fracture resistance in mandible and femur as well as improved the levels of calcium and phosphorous. However, studies are needed to determine if the improvement in bone biomechanical properties, as well as the mineral composition of the bone in experimental animals, retains long-term time after treatment with bisphosphonates drugs stopping.
